# Supplementary material for: “If my husband was in the labor ward with me, my baby wouldn’t have died”; experiences on birth companionship from a tertiary health facility, Tanzania
Source: PLoS One. 2025 Jan 9;20(1):e0309602. doi: 10.1371/journal.pone.0309602 (PMC11717287; doi:10.1371/journal.pone.0309602)
Supplement: Attachment — Submitted filename: ManuscriptRevision_PLOS.docx [file pone.0309602.s001.docx]

Summary of reviewer comments:

The comments below highlight several areas for improvement in the research paper. Notably, there are suggestions to enhance the clarity and specificity of the title, abstract, and introduction, emphasizing the importance of clearly defining the problem and its context. The methodology section needs revisions for better clarity, particularly in specifying the type of data collection used. The study setting details are deemed excessive, and the reviewer recommends focusing on essential information. Spelling and grammar issues throughout the document need addressing. In the results section, there are concerns about the placement and necessity of certain tables, as well as the need for more quotes and citations to support themes. The discussion section requires a more organized structure, critical analysis, and thorough exploration of previous research in Tanzania. Additionally, the reviewer suggests addressing limitations, providing practical implications, and discussing potential policy implications. Overall, there is a call for increased coherence, consistency, and attention to detail throughout the paper.

Discussion of specific areas for improvement:

- Introduction Clarity: The introduction requires restructuring to better articulate the problem from the outset. Beginning with a clearer delineation of the research problem, whether it be focused on respectful maternity care or broader maternal and fetal/neonatal outcomes, will provide readers with a more concise and engaging introduction.
- Methodology Section: The methodology section demands a thorough revision. The first sentence needs to be rewritten for better clarity and smoother sentence structure. Additionally, the type of data collection (interviews or focus groups) should be explicitly stated. The excessively detailed description of the study setting, including GPS coordinates and unrelated hospital information, should be streamlined for conciseness.
- Spelling and Grammar: A comprehensive review for spelling and grammar issues throughout the document is crucial. Instances such as the capitalized "focused" and unexplained acronyms like "FGD" and "IDIs" need correction for linguistic precision.
- Results Presentation: The placement and necessity of tables in the results section need justification. It is advisable to either integrate the information into the text or provide a brief explanation for the tables. Furthermore, there is a need for additional quotes and citations to substantiate the identified themes and sub-themes.
- Discussion Section Organization: The discussion section requires a more organized structure, including a clearer reintroduction and elaboration of the themes identified in the results. The reviewer also recommends a more critical analysis of the results, addressing limitations, and providing practical and policy implications. Additionally, exploring existing research in Tanzania and its relevance to the current study will enhance the depth of the discussion.

More specific comments:

- Title and Abstract: Consider revising the title to clearly indicate the study's methodology. Additionally, replace "Southern Highland" with descriptors like "rural" or "urban" for better reader comprehension.
- Abstract background: The abstract's background requires reworking to immediately engage the reader. Start by clearly defining the problem—whether it's focused on respectful maternity care or broader maternal and fetal/neonatal outcomes. Introduce why studying birth companionship is crucial in addressing these outcomes
- Abstract objective: Reconsider using "analyze" in the abstract objective. In qualitative studies, terms like "explore" or "examine" would align better with the research approach.
- Abstract methodology: Rewrite the first sentence of the abstract methodology for improved clarity and smoother sentence structure. Consider: "A qualitative exploratory approach was utilized for interviews with purposefully selected participants from September to October 2021."
- Abstract methodology: Clarify the type of data collection used (interviews or focus groups) for better understanding.
- Conduct a thorough check for spelling and grammar errors. Address the unnecessary capitalization of "focused" and clarify terms like "antenatal clinic."
- Abstract results: Avoid starting sentences with "But" for grammatical correctness. Revise sentence structure and spelling for improved readability.
- Introduction: Consider starting the introduction with paragraph 2, focusing on defining the problem from the outset. Major attention is needed for spelling and grammar throughout this section.
- Review references in paragraph 1. Avoid using "WHO recommends..." without referencing specific articles or texts from the WHO. Reframe sentences to refer to previous research.
- Introduction, paragraph 3. Ensure references are appropriately placed throughout. Provide clarity on the definition of labor and expand on factors influencing it.
- Introduction paragraph 5: Elaborate on how companionship can be influenced by various factors. Provide examples, such as age or religious background.
- Clarify the importance of healthcare providers' perceptions. Consider including a section on previous research detailing healthcare providers' views on companionship, especially in LMICs or sub-Saharan Africa.
- Introduction: Avoid unnecessary repetition of the WHO abbreviation in the introduction.
- Introduction: Clearly state the motivation behind the project. Specify the gap in previous research in Tanzania or sub-Saharan Africa concerning birth companionship and its effects on maternal outcomes. Where is the why? Why are you doing this project?
- Again reword your objective. Revise the objective to better align with the motivation for exploring the experiences of both pregnant women and healthcare providers. Clarify why both perspectives are essential and how they contribute to addressing potential miscommunication.
- Methods: Avoid reannouncing the aim in the methods section. Ensure adherence to PLOS instructions for the methods structure.
- Methods, study design: Justify the choice of an exploratory qualitative study and its alignment with the research objective. Provide information about the specific area in Tanzania.
- Methods, Study setting: Streamline the study setting section by omitting unnecessary details. Focus on essential information, such as the location in the south of Tanzania, and provide a clear rationale for choosing this zone.
- Methods: a map of the study setting could be useful instead of all of this text which is entirely unnecessary.
- Method, study setting: Trim down information on the hospital and focus on critical details, such as the number of women in the maternity ward, annual deliveries, and maternal outcomes.
- Method, study setting: Reposition the study setting objective, placing it more logically as the overall study objective, as it succinctly describes the research focus in Tanzania. “Therefore, this study aimed to explore desire, preferred childbirth companionship and perceived barriers among pregnant women and healthcare providers in Tanzania.”
- Methods, participants and recruitment: Streamline the participants and recruitment section. Clearly identify the roles of co-authors and provide concise details on the sampling strategies and reasons for participant recruitment.
- Methods: Provide clarity on the decision-making process regarding the exclusion of focus groups with healthcare providers.
- Methods: Condense the methods section, considering the inclusion of supplementary material or an appendix for excessive details.
- Method, data collection and analysis: again specify why co author did what for the in depth interviews.
- Method, data collection and analysis: Specify the roles of co-authors in in-depth interviews and focus groups. Describe how the interview guide for the interviews and focus groups was developed, whether within the research team or based on a framework. Justify and provide references for the in-depth interview and semi-structured guide.
- Method: Define acronyms like FGD and IDIs to enhance reader understanding.
- Method: Clarify the type of coding employed in the research—whether it was inductive or deductive.
- Results: Justify the placement of tables in the results section and ensure they are appropriately described or integrated into the text. Clearly indicate whether the themes apply to both women and healthcare providers.
- Results: Evaluate the necessity of Table 2 and, if retained, ensure it is justified and referenced appropriately.
- Results: Strengthen themes and subthemes with more quotes and citations to substantiate findings. Clarify the connection between subthemes and the overall theme. Are these themes for both the women and the healthcare providers. Very unclear. It almost seems like these could be two different studies? One looking at womens opinion and one at healthcare providers? Why combine? Please provide reasoning and justification? Some of themes or sub themes need more quotes or citations to back up the sub themes. As well it is unclear how the sub themes are connected to the overall theme that you described?
- Discussion: Revisit the first paragraph of the discussion to reintroduce and describe the themes again.
- Discussion (Previous Research in Tanzania): Enhance the discussion by incorporating more information about previous research in Tanzania, both qualitative and quantitative, on birth companionship and its impact on maternal outcomes.
- Discussion (Organization): Improve the organization of the discussion section by grouping related findings together for better flow and readability.
- Discussion (Citations): Verify the presence and appropriateness of citations in the discussion section.
- Discussion (Redundancy): Address redundancy in discussing the integration of study results with those of previous research.
- Discussion (Practical Implications): Include more discussion on the practical implications of the research and how the results contribute to the existing body of knowledge. Highlight what is novel in this research.
- Discussion (Critical Analysis): Move beyond summarizing findings and provide a more critical analysis of the results.
- Discussion (Limitations): Introduce a discussion of limitations and potential biases within the study.
- Discussion (Barriers to Companionship): Delve deeper into the barriers to implementing childbirth companionship. Provide detailed insights into challenges at both institutional and cultural levels.
- Discussion (Future Research): Conclude the discussion with suggestions for future research, emphasizing existing gaps in knowledge and proposing ways to address them.
- Discussion (Consistency): Ensure consistency in language and style throughout the discussion. Avoid interchanging terms like "companionship" and "companion."
- Discussion (Policy Implications): Discuss potential policy implications based on the findings. Outline how healthcare policies could be shaped or modified to accommodate preferences and needs identified in the study.
