# Supplementary material for: “If my husband was in the labor ward with me, my baby wouldn’t have died”; experiences on birth companionship from a tertiary health facility, Tanzania
Source: PLoS One. 2025 Jan 9;20(1):e0309602. doi: 10.1371/journal.pone.0309602 (PMC11717287; doi:10.1371/journal.pone.0309602)
Supplement: Attachment — Submitted filename: PONE-D-23-19544_reviewer.docx [file pone.0309602.s002.docx]

Authors present a pertinent study for women healthcare in Tanzania. It is interesting to have women voices as well as health care professionals, even more when they both conclude the need of strengthening companionship during labor in the study setting.

Mayor revision

Denial of companionship in labor is  [a manifestation of obstetric violence](https://www.tandfonline.com/doi/full/10.1080/26410397.2021.2004634) that enhances the possibility of other mistreatments during labor [(e.g.](https://link.springer.com/article/10.1186/s12884-023-05811-2) or [e.g.2)](https://www.sciencedirect.com/science/article/abs/pii/S0266613823001699). This is important to address in introduction, results and discussion as there are important results that indicate obstetric violence lived by participants in the study setting.

Revisions in each section of the report:

**Abstract**

1. Simplify introduction: Consider to start the paragraph with the third phrase and to merge two first sentences. Include a phrase resuming the justification of the study in the context of Tanzania (e.g. even though there are politics to enhance companionship, it is not being very common).
2. Key words: consider to include standardized terms (e.g. maternal healthcare) and exclude “desire” as it is unspecific to the study

**Introduction**

3. The authors develop a precise overview of childbirth companionship and maternal healthcare. A focus on local context on the matter needs to be given in introduction (maternal healthcare and companionship in Tanzania; socio economic differences between hospitals, cultural / religious context) that may influence the phenomenon.
4. p. 4. Interesting evidence around preferences of women companionships, influenced by education and beliefs. Please describe examples of these factors.

**Methodology**

**Study setting**

5. Please clarify socioeconomic conditions of patients attended in the selected hospital.
6. Revise the last phrase that presents the purpose of the study. Consider to correct by excluding the word “desire”.

**Participants:**

7. Good rationale related to sampling. Revise writing to gain clarity in this procedure.
8. The first time to include an acronym, also include the words being summarized.
9. It is necessary to explain sampling/recruitment procedure with the mothers` group.
10. Include a reference to Table 1.

**Data collection:**

11. Please explain if in the focus groups design authors considered any segmentation criteria to group participants.

**Analysis:**

12. Please separate this section from data collection.

**Trustworthiness:**

13. Please clarify the acronyms IDI and FGD. If a maximum variation sampling was performed, it should also be described in the participants section along with the chain strategy described.
14. As Authors developed initial coding individually, you can also declare triangulation as a trustworthiness strategy.
15. There is a need of positionality and reflexivity processes declarations of the authors as physicians. ¿Do authors work in the institution? this is also important to be transparent with possible interests in conflict in the study. ¿Are participants also their patients?

**Results**:

16. Table 1. Please clarify what does Standard 7 (education) for international audience. what are the health professions included in the interviews?
17. The paragraph below table 2 can be removed as it presents the same information of the table. Authors may consider instead a brief resume of the particular differences on the results between the two groups.
18. The theme “creation of close relationships in the family” seems to be related to the enhancement of women health, by reducing gender violence and inequity from the perspective of women and health professionals. This is an important result that deserves to be highlighted.
19. Theme “mother having a sense of security”: The strong institutional and interpersonal violence experienced by the woman participant during labor needs to me addressed by authors in their description of the theme. The four “Ps” described in results seems to be part of the discussion. Something similar is found in “society of fear”. Remember to focus on results expressed by participants in this section.
20. Theme: “Staffs are avoiding accountability for their shortcomings”: It is important to clarify this theme and revise the quote writing and punctuation to gain clarity. Is this something said only by the staff?
21. Theme “culture of covering mistakes”: The authors description and participant quote evidence a culture of covering gender violence, particularly during labor. Not simple “mistakes”. evidence reported in previous theme also showed gender violence in women healthcare service during labor. This is highly important and should be evident in the name of the theme: it enhances the relevance of their companionship, and shows institutional and structural barriers to the phenomenon under study.

**Discussion and conclusion:**

22. This section can be strengthened framing the results in the global aim of a respected childbirth healthcare. The consulted [WHO guidelines to enhance childbirth companionships](https://iris.who.int/bitstream/handle/10665/334151/WHO-SRH-20.13-eng.pdf?sequence=1) can also guide discussion related to the identified barriers.
23. Results related to obstetric violence should be discussed in this section.
24. Please frame conclusion in the context and scope of the study.

**Data availability:** please clarify where or how is the data available.

**Review English writing:**

25. Please review English writing. There are multiple semicolons that might not be appropriate in the paragraph.
26. Introduction: first paragraph (The who recommends…; However, in real clinical situations). Also review wording of third paragraph, first phrase.
27. p.6. “Mbeya zonal referral hospital has obstetrics and gynecology department which saves the people from different parts of Mbeya city and Southern highland zone with complicated obstetric and gynecological conditions” (Do author mean “attends”?)
28. p. 7. “and focused group discussion ~~to the~~ with pregnant women”
