# Supplementary material for: “If my husband was in the labor ward with me, my baby wouldn’t have died”; experiences on birth companionship from a tertiary health facility, Tanzania
Source: PLoS One. 2025 Jan 9;20(1):e0309602. doi: 10.1371/journal.pone.0309602 (PMC11717287; doi:10.1371/journal.pone.0309602)
Supplement: Attachment — Submitted filename: matrix_response.docx [file pone.0309602.s003.docx]

**RESPONSE TO REVIEWER’S COMMENTS**

| **S/N** | **Section** | **Comments from the reviewers** | **PI’s response** |
| --- | --- | --- | --- |
| 1 | **Title** | Southern highland | Removed |
| 2 | **Abstract** | Background requires reworking to immediately engage the reader | Background – restructured |
|  |  | Use of the word analysis | Changed to explore |
|  |  | Methodology | Restructured |
|  |  | Structure and spelling | Recorrected |
| 3 | **Introduction** | Reaarrangement of the paragraphs | Reaaranged |
|  |  | Grammar and spelling | Recorrected |
| 4 | **Methodology** | Study design | Restructured |
|  |  | Study setting | Number of deliveries per month  Map of Southern highland zone added |
|  |  | Restructuring methodology | Restructured |
| 5 | **Results** | Table of demographic data | Summarized in words |
|  |  | Restructure the themes | Restructured |
| 6 | **Discussion** | Restructuring in regard with findings | Restructured |
|  |  | Previous Research in Tanzania | With my best, I have found only one study conducted in Tanzania |
| 7 | **General Comments** | Grammar and English writing | Recorrected |
