# Supplementary material for: “If my husband was in the labor ward with me, my baby wouldn’t have died”; experiences on birth companionship from a tertiary health facility, Tanzania
Source: PLoS One. 2025 Jan 9;20(1):e0309602. doi: 10.1371/journal.pone.0309602 (PMC11717287; doi:10.1371/journal.pone.0309602)
Supplement: Attachment — Submitted filename: Michael - Reviewers Responses.docx [file pone.0309602.s004.docx]

| **Reviewer’s comment** | **Author’s response** | **Page and lines the changes inserted** |
| --- | --- | --- |
| **Title** | | |
| Reviewer #1  Replace "Southern Highland" with descriptors like "rural" or "urban" for better reader comprehension.  Reviewer #2  No comment | Removed Southern highland for better reader comprehension  Thank you | 1 |
| **Abstract** | | |
| Reviewer#1   - Abstract background: The abstract's background requires reworking to immediately engage the reader. - Abstract objective: Reconsider using "analyze" in the abstract objective - Abstract methodology: Rewrite the first sentence of the abstract methodology for improved clarity and smoother sentence structure - Abstract methodology: Clarify the type of data collection used (interviews or focus groups) for better understanding. - Conduct a thorough check for spelling and grammar errors - Abstract results: Avoid starting sentences with "But" for grammatical correctness. Revise sentence structure and spelling for improved readability.   Reviewer #2  - Simplify introduction  - Key words | -Restructured and focused on maternal and foetal outcome  -Explore was used instead of Analyse  -The sentence was rewritten  -Both interviews and focused group discussion Rewritten  -Spelling and grammar errors corrected  -Sentence revised and corrected for better readability  - Background simplified  - Standardized terms included | 2 |
| **Introduction** | | |
| Reviewer #1   - Consider starting the introduction with paragraph 2 - Major attention is needed for spelling and grammar throughout this section - Review references in paragraph 1. - Paragraph 3. Ensure references are appropriately placed throughout. Provide clarity on the definition of labor and expand on factors influencing it. - Elaborate on how companionship can be influenced by various factors. - Avoid unnecessary repetition of the WHO abbreviation in the introduction - Clearly state the motivation behind the project   Reviewer #2   - The authors develop a precise overview of childbirth companionship and maternal healthcare - p. 4. Interesting evidence around preferences of women companionships, influenced by education and beliefs. | -Started with paragraph 2  -Spelling and grammar rechecked throughout  -References reviewed and corrected – currently paragraph 2  -Labor and factors influencing it well clarified  -References rechecked and corrected  -Physical environment and sociocultural norms are key factors influencing companionship  - Corrected  -Last paragraph is the motivation statement behind the project.  -Thank you for your appreciation  - Few local studies done  -Thank you for your appreciation.  Orthodox religion and Yoruba tribe in Nigeria are examples | 3-5 |
| **Methodology** | | |
| Reviewer #1   - Avoid reannouncing the aim in the methods section. Ensure adherence to PLOS instructions for the methods structure - Justify the choice of an exploratory qualitative study and its alignment with the research objective. Provide information about the specific area in Tanzania. - Streamline the study setting section by omitting unnecessary details - A map of the study setting could be useful instead of all of this text which is entirely unnecessary. - Trim down information on the hospital and focus on critical details. - Reposition the study setting objective, placing it more logically as the overall study objective, as it succinctly describes the research focus in Tanzania - Streamline the participants and recruitment section. - Clearly identify the roles of co-authors. - Provide clarity on the decision-making process regarding the exclusion of focus groups with healthcare providers - Condense the methods section - Describe how the interview guide for the interviews and focus groups was developed, whether within the research team or based on a framework - : Clarify the type of coding employed in the research   Reviewer #2   - Please clarify socioeconomic conditions of patients attended in the selected hospital - Revise the last phrase that presents the purpose of the study - Good rationale related to sampling - The first time to include an acronym, also include the words being summarized - It is necessary to explain sampling/recruitment procedure with the mothers` group. - Include a reference to Table 1 - Please explain if in the focus groups design authors considered any segmentation criteria to group participants - Please separate this section from data collection - As Authors developed initial coding individually - Do authors work in the institution | -Thank you.  Aim of the study removed.  -To my level best justified  -Thank you. Unnecessary details omitted  -Thank you. A map of the study setting added  -Thank you, Done  -Thank you. Done  -Thank you. Done  -Role of each author clarified  -Shortage of staff and tight timetable were the barriers to FGD  -Thank you. Done  -Interview guide was developed within the research team  -Inductive coding  -Thank you. Socioeconomic clarified.  -Thank you. Revised and corrected  -Thank you.  -Thank you. Rechecked  -Thank you. Sampling explained  -Table 1 summarize in words  -In focus group, segmentation criteria was not considered  -Thank you. Data collection Separated with data analysis  - Thank you. Initial coding done in group of 2 authors  - Only Principal author work in the institution | 6-10  21 |
| Results | | |
| Reviewer #1   - Justify the placement of tables in the results section and ensure they are appropriately described or integrated into the text. Clearly indicate whether the themes apply to both women and healthcare providers. - Strengthen themes and subthemes with more quotes and citations to substantiate findings   Reviewer #2   - Table 1. Please clarify what does Standard 7 (education) for international audience - What are the health professions included in the interviews? - The paragraph below table 2 can be removed as it presents the same information of the table - The theme “creation of close relationships in the family” seems to be related to the enhancement of women health, by reducing gender violence and inequity from the perspective of women and health professionals - Theme “mother having a sense of security”: The strong institutional and interpersonal violence experienced by the woman participant during labor needs to me addressed by authors in their description of the theme. The four “Ps” described in results seems to be part of the discussion. Something similar is found in “society of fear”. Remember to focus on results expressed by participants in this section. - Theme: “Staffs are avoiding accountability for their shortcomings”: It is important to clarify this theme and revise the quote writing and punctuation to gain clarity. Is this something said only by the staff? - Theme “culture of covering mistakes”:  The authors description and participant quote evidence a culture of covering gender violence, particularly during labor. Not simple “mistakes”.  evidence reported in previous theme also showed gender violence in women healthcare service during labor. This is highly important and should be evident in the name of the theme: it enhances the relevance of their companionship, and shows institutional and structural barriers to the phenomenon under study | -Thank you. Tables summarized in words  -Themes and sub-themes apply to both pregnant women and healthcare providers  -Thank you. To our level best, some modifications done.  -Thank you. Standard 7 is primary education. Changed  -Doctors and nurses were interviewed  -Thank you. Table 2 removed  -Thank you. Corrected as you advised.  - Thank you. Corrected as you advised.  - Thank you. Corrected as you advised.  -Thank you. Corrected as you advised. | 11-15 |
| **Discussion** | | |
| Reviewer #1   - Revisit the first paragraph of the discussion to reintroduce and describe the themes again. - Previous Research in Tanzania - Improve the organization of the discussion section by grouping related findings together for better flow and readability - Verify the presence and appropriateness of citations in the discussion section. - Include more discussion on the practical implications of the research and how the results contribute to the existing body of knowledge. Highlight what is novel in this research. - Move beyond summarizing findings and provide a more critical analysis of the results. - Discussion (Barriers to Companionship) - Discussion (Future Research) - Discussion (Consistency) - Discuss potential policy implications based on the findings   Reviewer #2   - This section can be strengthened framing the results in the global aim of a respected childbirth healthcare - Results related to obstetric violence should be discussed in this section - Please frame conclusion in the context and scope of the study | -Thank you. Revisited and corrected.  -Two studies from Tanzania included in my discussion  -Thank you. Reorganized for better flow and readability.  -Thank you. Rechecked and corrected  -Thank you. Practical implication included  -Thank you. To our level best critically analysed  -Thank you. Discussed  -Thank you. Added and discussed  -Thank you. Rewritten and consistency considered  -Thank you. Discussed to our level best.  -Thank you. The section strengthened  -Thank you. Discussed  -Thank you. Conclusion framed | 16-18 |
| **Data availability:**  please clarify where or how the data is available. | -Thank you. Data availability rewritten | 20 |
| **Review English writing:** | -Thank you. English writing reviewed the whole document | N/A |
