# Supplementary material for: “If my husband was in the labor ward with me, my baby wouldn’t have died”; experiences on birth companionship from a tertiary health facility, Tanzania
Source: PLoS One. 2025 Jan 9;20(1):e0309602. doi: 10.1371/journal.pone.0309602 (PMC11717287; doi:10.1371/journal.pone.0309602)
Supplement: Attachment — Submitted filename: Reviewer Responses -2.docx [file pone.0309602.s006.docx]

| **Reviewer’s comment** | **Author’s response** | **Page and lines the changes inserted** |
| --- | --- | --- |
| **Title** | |  |
| heath | health | 1 |
| **Introduction** | | **3 - 5** |
| 1. How many or what proportion? 2. Reference 3. In what context? 4. Seem to have? What does the data say? 5. This repeat what you said in par 1 6. Revise English 7. it – what? 8. Unnecessary information 9. Revise English. Use shorter sentences. 10. Where is reference 28? 11. RMNCAH – what does it mean? 12. What studies? Add the references 13. Revise english | - Changed the word majority to most  -Reference added  - Changed to most recently reference and paraphrased  - Thank you. English have been corrected  - Thank you. Deleted  - Thank you. English revised  - Thank you. companionship  - Thank you. Deleted  - Thank you. Information summarized  - Thank you. References crosschecked  - Thank you. Defined  - Thank you. References added  - Thank you. English Revised |  |
| **Materials and Methods** | | **6 - 11** |
| 15. Provide a reference 16. Be consistent with the use of acronyms 17. What do you mean? 18. Indicate where to insert the figure and its title. Also, you need to explain the meaning of the colours on the map. Consider offering an additional map to indicate the location of this area in relation to the country. 19. Reference to these techniques? 20. Later in the text, you say you used maximum variation sampling. Please be consistent and clarify the difference 21. How many years? Less than 4 years and more than 6 months? Please clarify 22. Participants and recruitment - part 23. Data collection - part 24. Data Processing and analysis 25. Methodological consideration | - Thank you. Reference provided  - Thank you  - Thank you. Paraphrased and unnecessary information deleted  - Thank you. Map changed  - Thank you. Reference added  - Thank. Corrected to purposive sampling  -Thank you. Rewritten and Clarified  - Thank you. Some of the information deleted and restructured  - Thank you. Collected and rearranged as you advised.  -Thank you. Changed and clarified as you advised.  - Thank you. Changed and deleted unnecessary information. |  |
| **Results** | | 12 - 16 |
| 26. blames- blame 27. psych –what is this? | -Thank you. Changed to blame  -Thank you. Changed to psyche |  |
| **Discussion** | | 17 - 18 |
| 28. Why? You did not address the issues about policies, lack of capacity or community participation. | -Thank you. This part deleted  - Also unnecessary information deleted as you advised |  |
| **Conclusion** | | 19 |
| 29. Delete, this sentence is not a conclusion. 30. You should not repeat the results in the conclusions section 31. Although this might be correct, it is not a conclusion of your study. | -Thank you. The sentence deleted  -Thank you. The information deleted  -Thank you. The information deleted |  |
